# Rhamnolipid from *Pseudomonas* sp. as a green surfactant for enhanced phytoremediation

**DOI:** 10.1038/s41598-025-14244-0

**Published:** 2025-08-14

**Authors:** Ahmed Sorour, Najlaa Zobair, Khaled Ghanem, Heba Khairy

**Affiliations:** Department of Botany and Microbiology, Faculty of Science, Alexandria University, Moharam Bek, Alexandria, 21511 Egypt

**Keywords:** *Pseudomonas* sp., Rhamnolipids, Biosurfactant-assisted phytoremediation, *HaZIP*, *Helianthus annus*

## Abstract

Microbial biosurfactants are valued for their surface activity and emulsifying properties; among them, rhamnolipids—primarily produced by *Pseudomonas* species—are the most prominent. *Pseudomonas* sp., a plant growth-promoting rhizobacterium, is also known to enhance heavy metal (HM) uptake in *Helianthus annuus* L. In this study, we produced biosurfactants from *Pseudomonas aeruginosa* strain ZF2MGHSO (Rha1) and *Pseudomonas sp.* strain AHE16 (Rha2). Gas chromatography−mass spectrometry (GC–MS) analysis confirmed that the purified biosurfactant was composed of rhamnolipids. We evaluated the effects of Rha1 and Rha2 on Cd and Zn uptake and *HaZIP1* gene expression in sunflower plants grown in contaminated soil. Both rhamnolipids significantly increased Zn and Cd accumulation in roots and shoots, with the highest root Zn (724 ± 3 mg g⁻^1^ DW) and Cd (173 ± 2 mg g⁻^1^ DW) levels recorded in Rha1-treated plants. In shoots, Zn concentrations reached 460 ± 4 mg g⁻^1^ DW with Rha1 and 426 ± 3 mg g⁻^1^ DW with Rha2, compared to 405 ± 3 mg g⁻^1^ DW in control. The relative expression of *HaZIP1* was significantly upregulated in both roots and shoots under rhamnolipid treatments. In Rha1-treated plants, expression levels increased ~ 6.9-fold in roots and ~ 4.8-fold in shoots compared to control. Rha2 treatment led to ~ 6.0-fold and ~ 4.1-fold increases in roots and shoots, respectively. Our findings suggest that *HaZIP1* plays a pivotal role in the uptake and accumulation of zinc and cadmium in sunflower plants grown in contaminated soil. Overall, our study highlights the potential of biosurfactant-enhanced phytoremediation using sunflower plants as an efficient, environmentally sustainable strategy for remediating heavy metal-contaminated soils.

## Introduction

Rhamnolipids, a class of biosurfactants initially described as oily glycolipids^1^, were introduced to the market a decade ago and have since found numerous industrial applications. *Pseudomonas aeruginosa* and several *Burkholderia* species produce them naturally^2^. Since rhamnolipid synthesis and regulation are important virulence factors in *P. aeruginosa*, they have been examined in detail^3^. In human respiratory epithelium, for instance, it has been shown that rhamnolipids decrease mucociliary transport^4^. It has also been shown to be implicated in biofilm formation^5^ and swarming motility^6^. Moreover, many studies reported that *P. aeruginosa* produced rhamnolipid, which had excellent physicochemical qualities and might be employed in a variety of industrial products^7,8^. In comparison to other bacterial biosurfactants, they exhibit minimal toxicity^9^, and good biodegradability^2^. While *P. aeruginosa* is a prolific biosurfactant producer, surfactin from *Bacillus subtilis* can also be synthesized efficiently under optimized conditions^10^.

The majority of *P. aeruginosa* strains produce two types of rhamnolipids: di-rhamnolipid, which contains two rhamnose molecules and a fatty acid dimer, and mono-rhamnolipids, which have one rhamnose moiety and a fatty acid dimer. Mono-rhamnolipid (mono-Rha) synthesis is mediated by the coordinated activity of RhlA (3-hydroxyacyl-ACP transferase), which catalyzes the formation of a fatty acid dimer using a CoA-linked fatty acid derivative produced by RhlY (enoyl-CoA hydratase) and RhlZ (enoyl-CoA hydratase/isomerase)^11,12^. This dimer, along with dTDP-L-rhamnose (thymidine diphosphate L-rhamnose), an activated sugar donor derived from dTDP-glucose, serves as a substrate for RhlB (rhamnosyltransferase), facilitating mono-Rha biosynthesis. Similarly, RhlC (rhamnosyltransferase 2) catalyzes the conversion of mono-Rha into di-rhamnolipid (di-Rha) by utilizing mono-Rha and dTDP-L-rhamnose as substrates^2^. Furthermore, *Pseudomonas* sp. strain AHE16 accession number OL862991.1 was identified among plant growth-promoting rhizobacteria (PGPR) that enhance heavy metal uptake and accumulation in *Helianthus annuus* L. plants via phosphate solubilization and Indole-3-Acetic Acid (IAA) Production^13^.

The contamination of agricultural soils with heavy metals and metalloids (HMs) is a major environmental challenge, degrading soil health, reducing crop productivity, and posing risks to food safety^14^. To mitigate these effects, microbial surfactants such as rhamnolipids and surfactin enhance metal bioremediation by increasing heavy metal bioavailability through solubilization and desorption^15,16^. Additionally, phytoremediation, including phytoextraction and phytostabilization mechanisms, is a sustainable and eco-friendly strategy for remediating heavy metal-contaminated sites^13,17^.

Sunflower (*Helianthus annuus* L.) is a promising candidate for phytoremediation due to its ability to accumulate heavy metals from contaminated soils. The Egyptian cultivar V120, in particular, has shown potential for efficiently removing cadmium (Cd) and zinc (Zn), making it a valuable species for restoring metal-contaminated environments and improving soil quality^18^.

Successful phytoremediation relies on specialized metal transport mechanisms that regulate metal ion uptake, translocation, and homeostasis in plants. Among these, the Zinc-regulated, Iron-regulated transporter-like Protein (ZIP) family plays a key role in metal acquisition and distribution. Initially discovered in *Arabidopsis thaliana*^19^, ZIP transporters in tobacco have been shown to mediate Zn uptake, long-distance transport, and metal homeostasis^20^. Their roles in phytoremediation, particularly in metal accumulation, are well-established. In addition to Zn^2^⁺, ZIP transporters facilitate the transport of other metal cations, including manganese (Mn^2^⁺), iron (Fe^2^⁺), cadmium (Cd^2^⁺), cobalt (Co^2^⁺), copper (Cu^2^⁺), and nickel (Ni^2^⁺)^21,22^.

This study aimed to assess the effects of two biologically produced rhamnolipids on heavy metal uptake, accumulation, and the expression of the *Helianthus annuus* *ZIP1* (*HaZIP1*) transporter gene. The present work involved producing and characterizing a biosurfactant from *Pseudomonas* sp. strain AHE16 (Rha2) and comparing it with a previously produced rhamnolipid from *Pseudomonas aeruginosa* strain ZF2MGHSO (Rha1). The primary goal was to investigate rhamnolipid-assisted phytoremediation, and the underlying mechanisms of heavy metal homeostasis, with a particular focus on the role of the ZIP1 transporter.

## Results

### Biosurfactant production and evaluation

The *Pseudomonas* species used in this study were cultured for biosurfactant production in a medium supplemented with previously used vegetable oil as the sole carbon and energy source.

#### Emulsification index of cell-free supernatant (CFS)

The emulsification index of the biosurfactant in the CFS produced by *Pseudomonas* sp. was tested against engine oil, xylene, benzene, and diesel oil (Table 1). The results indicated that both the strains exhibited highest emulsification index against benzene mainly with E_24_ up to 80%. While slight emulsifying index was detected against both diesel oil and xylene.

**Table 1:** Emulsification index of cell-free supernatants from *Pseudomonas* sp. strain AHE16 and *Pseudomonas aeruginosa* strain ZF2MGHSO grown in MSM supplemented with used vegetable oil as a sole carbon source. Data are means ± SD of (*n* = *3*).

Hydrocarbons	Emulsification index E_24_ (%)	
	AHE16	ZF2MGHSO
Diesel oil	3.33 ± 0.32	2.5 ± 0.51
Engine oil	55.23 ± 1.75	52 ± 2.85
Xylene	5.47 ± 0.62	7.7 ± 0.92
Benzene	77.25 ± 2.5	80 ± 2.34

#### Oil displacement assay

**Fig. 1:**
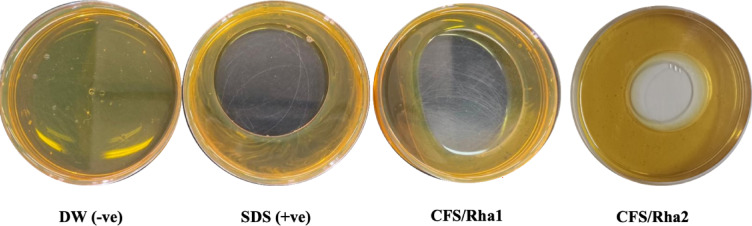
Oil displacement assay. Cell free supernatant containing rhamnolipids produced from *Pseudomonas aeruginosa strain* ZF2MGHSO (Rha1) and *Pseudomonas* sp. strain AHE16 (Rha2). SDS: sodium dodecyl sulfate as a positive control, 1% (w/v), distilled water (DW): as a negative control.

The surface activity of biosurfactant in the CFS was directly proportional to the diameter of the clear zone detected. As shown in Fig. 1, both CFS showed promising and potential biosurfactant activity. As the oil displacement zone diameter for both strains were about 5 to 5.5 cm, while its area ranged from 20 to 23.7 cm^2^ which was comparable to that recorded by SDS as a positive control.

#### Parafilm M test

**Fig. 2:**
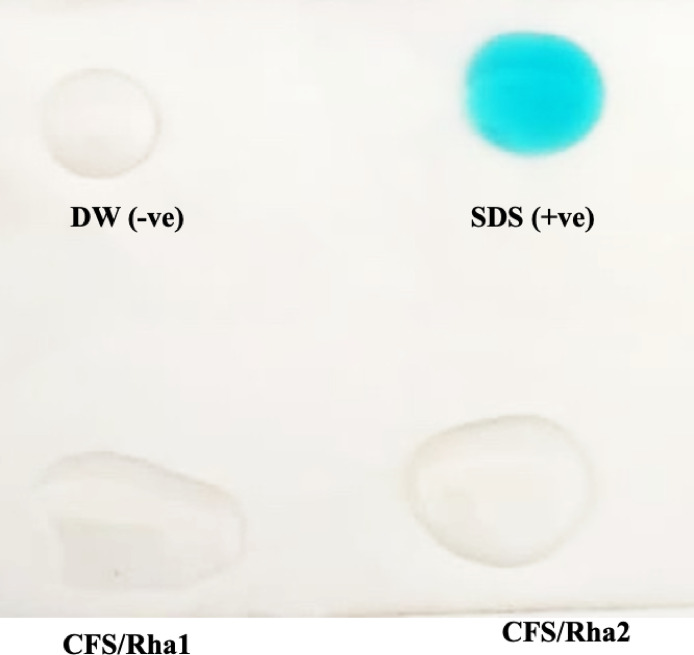
Parafilm M test. Cell free supernatant containing rhamnolipids produced from *Pseudomonas aeruginosa strain* ZF2MGHSO (Rha1) and *Pseudomonas* sp. strain AHE16 (Rha2). SDS: sodium dodecyl sulfate as a positive control, 1% (w/v), distilled water (DW): as a negative control.

The Parafilm M test was utilized as a preliminary screening method to assess biosurfactant production. The results revealed that the droplet diameters of cell-free supernatants from both *Pseudomonas* sp. were larger than those of H_2_O (negative control), as shown in Fig. 2. Revealing the nature of biosurfactant, CFS of both isolates deemed to be positive producers (with a score ≥ ++) when applying CFS droplets on parafilm.

### Extraction, purification and characterization of biosurfactant

In previous experiments, the use of waste vegetable oil as a carbon source resulted in significantly higher biosurfactant production (manuscript under review). Consequently, used vegetable oil was selected as the carbon source for this study to investigate and compare biosurfactant production from PGPR *Pseudomonas* sp. strain AHE16^13^ with our previously isolated bacterium *Pseudomonas aeruginosa* strain ZF2MGHSO.

**Fig. 3:**
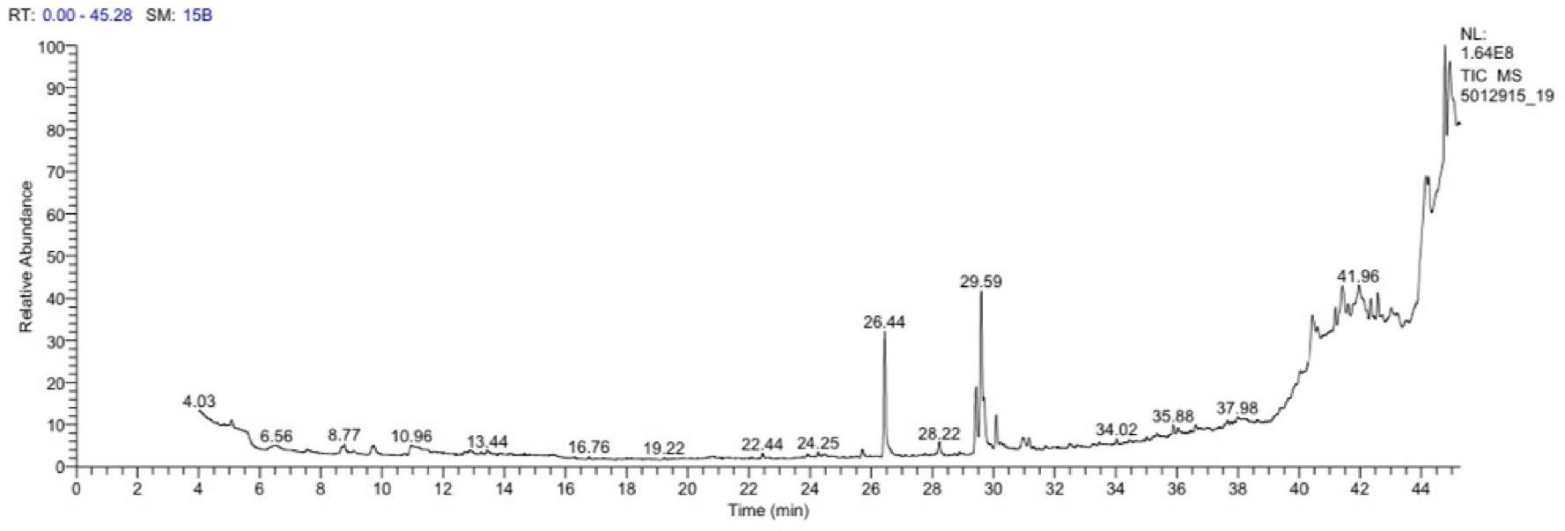
GC–MS spectrum of the biosurfactant produced by *Pseudomonas* sp*.* strain AHE16 grown in mineral salt medium supplemented with 2% used vegatble oil as carbon source.

After purifying biosurfactant produced by strain AHE16 it was analyzed using GC–MS (Gas Chromatography–Mass Spectrometry). The GC–MS chromatogram of the produced biosurfactant revealed eight distinct peaks, indicating the presence of various compounds (Fig. 3). The major compounds were identified at retention times (RT) of 22.44, 24.25, 26.44, 35.88, and 41.96 min, corresponding to tetradecanoic acid, 9-Octadecen-1-ol, (z)-, n-Hexadecanoic avid, Bis(2-ethylhexyl) phthalate, and 2,4-Di-tert-butylphenol, respectively. Additionally, our rhamnolipid’s mass spectrum patterns indicate that it is formed of mono-rhamnolipid structure: Rha-C10. The mono-rhamnolipid’s fatty acid component, on the other hand, was made up of three saturated hydroxy fatty acids: 14, 16 and 18. Finally, our biosurfactant was named as Rha2 and used for further experiments.

### Rhamnolipid-assisted Phytoremediation

#### Biosurfactant-assisted heavy metal uptake and accumulation in *Helianthus annuus*

**Fig. 4:**
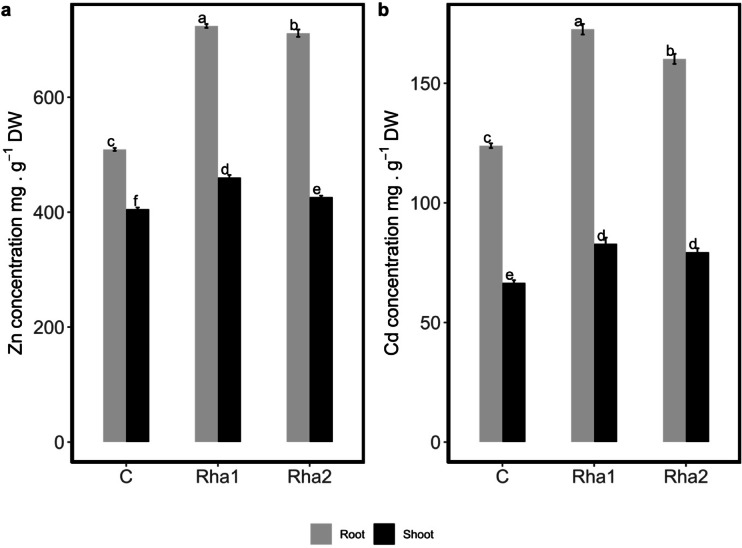
Heavy metal concentrations in roots and shoots of *H. annuus* plants grown in the experimental soil with two rhamnolipids (**a**) Zinc and (**b**) cadmium. Different letters in each column indicate significant differences between means ± SD of treatments (*n* = *3*) at a *P* < *0.05* significance level according to Tukey’s posthoc tests for multiple comparisons of means following two-way ANOVA.

Both rhamnolipids did not show inhibitory effect on either seed germination or root growth (data not shown). Zinc and cadmium concentrations in plant shoots and roots were determined and expressed as mg g⁻^1^ dry weight (DW). At the end of the growth period, Zn and Cd concentrations were significantly higher in roots and shoots of plants grown on experimental soil irrigated with the two rhamnolipids (Rha1 and Rha2) compared to plants grown on experimental soil without surfactants (C) (Fig. 4). Furthermore, plants treated with Rha1 showed a higher root accumulation of Zn and Cd than plants treated with Rha2. In more detail, with Rha1, *Helianthus annus* roots accumulated higher concentrations of zinc (724 ± 3 mg g^−1^ DW) and cadmium (173 ± 2 mg g^−1^ DW), compared to lower values (711 ± 6 and 160 ± 2 mg g^−1^ DW) recorded with Rha2 for zinc and cadmium, receptively. Generally, significantly higher accumulations of Zn and Cd were observed in shoots of plants grown on experimental soil with Rha1 and Rha2 compared to untreated plants used as control. On soil with rhamnolipids, plant shoots accumulated significantly higher concentrations of zinc (460 ± 4 and 426 ± 3 mg g^−1^ DW) for Rha1 and Rha2, respectively, compared to 405 ± 3 mg g^−1^ DW recorded in shoots of control plants. Furthermore, treated plant shoots exhibited significantly enhanced cadmium accumulation, 1.2-fold higher, compared to control. In contrast, there were no significant differences between the two rhamnolipids in the accumulation of Cd in shoots of plants grown in experimental soil.

#### Changes in the expression of *ZIP1* transporter gene

**Fig. 5:**
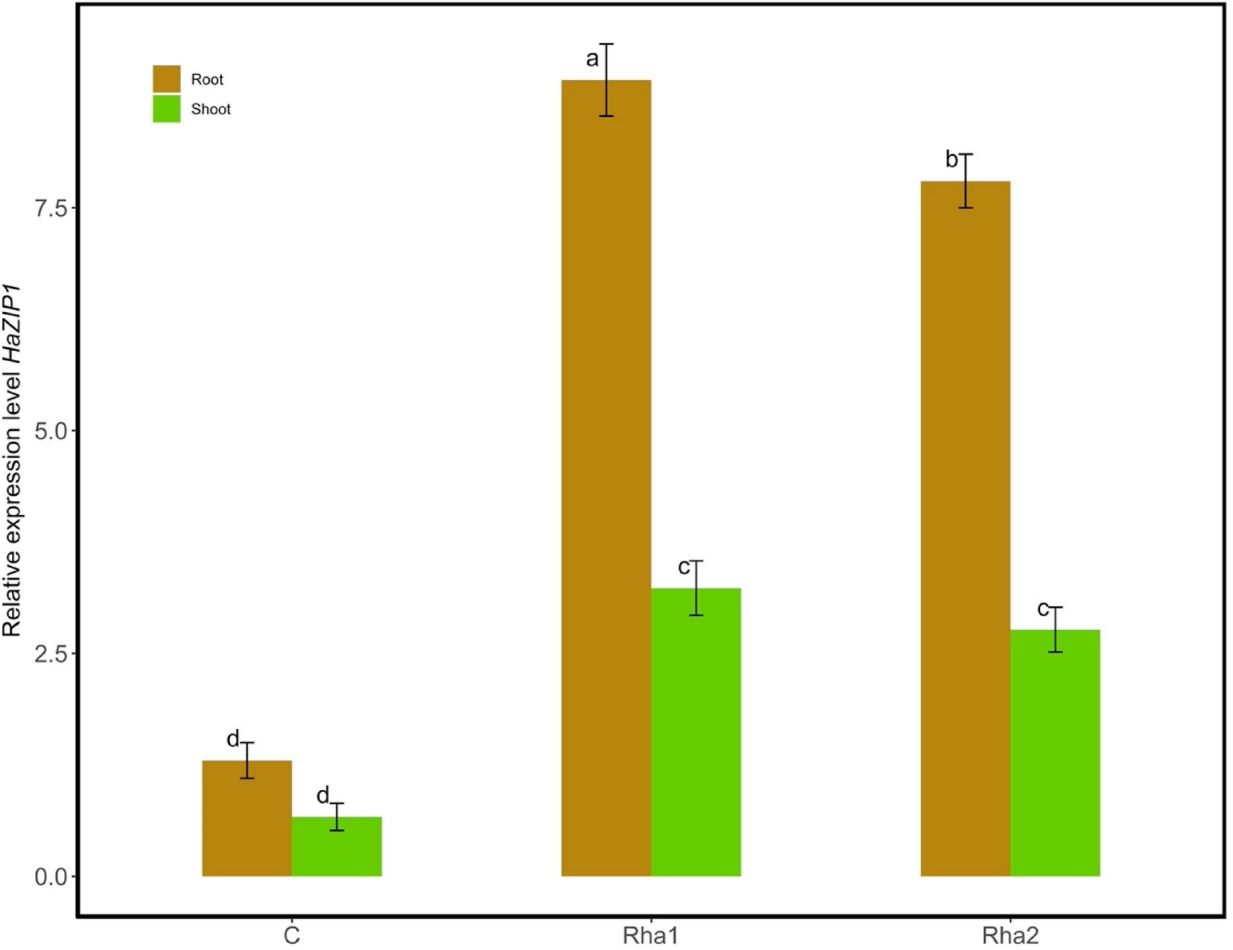
Relative expression levels of *HaZIP1* in roots and shoots of *H. annuus* plants grown in the experimental soil with two rhamnolipids. Transcript levels were normalized to the reference gene *EF1α* and are expressed as fold changes. Different letters in each column indicate significant differences between means ± SD of treatments (*n* = *3*) at a *P* < *0.05* significance level according to Tukey’s posthoc tests for multiple comparisons of means following two-way ANOVA.

The relative expression of *HaZIP1*, normalized to the reference gene *EF1α*, did not show a significant difference between the roots (1.30 ± 0.20) and shoots (0.67 ± 0.15) of control plants (Fig. 5). However, in plants grown in experimental soil treated with rhamnolipids (Rha1 and Rha2), *HaZIP1* expression was significantly upregulated in both roots and shoots compared to control. In Rha1-treated plants, *HaZIP1* expression reached 8.93 ± 0.40 in roots and 3.23 ± 0.31 in shoots, corresponding to approximately 6.9-fold and 4.8-fold increases over the respective control tissues. Similarly, Rha2 treatment led to expression levels of 7.80 ± 0.30 in roots and 2.77 ± 0.25 in shoots—representing 6.0-fold and 4.1-fold increases, respectively.

In both treatments, roots exhibited significantly higher expression than shoots, with root expression being approximately 2.8-fold greater than shoot expression for both Rha1 and Rha2. These results suggest a stronger transcriptional response to rhamnolipid treatment in root tissues and a potential role of *HaZIP1* in mediating enhanced metal uptake under these conditions.

## Discussion

*Pseudomonas* sp., particularly *Pseudomonas aeruginosa*, are widely encountered Gram-negative environmental bacteria known for their metabolic versatility and ability to colonize diverse habitats. *P. aeruginosa* is a paradoxical microorganism; while it poses significant health risks as an opportunistic pathogen, it also produces valuable biosurfactants with important industrial and environmental applications^23,24^. Effective management strategies are crucial to maximizing its benefits while minimizing its risks. Despite its potential hazards, controlled fermentation techniques enable the safe utilization of *P. aeruginosa* for biosurfactant production.

However, the specific role of these biosurfactants within bacterial cells remains unclear. Suggested physiological functions include the solubilization of insoluble substrates, adhesion to interfaces in natural ecosystems, and metal complexation. These functions may serve as adaptive strategies that enhance microbial survival in metal-rich environments by reducing or inhibiting cellular toxicity^24^. In a-comparative study of two *Pseudomonas* sp. isolated from different niches in their ability to produce biosurfactant, we observed that both strains were able to produce bioactive compounds showing biosurfactant properties. In the current study, *Pseudomonas aeruginosa* strain ZF2MGHSO produced rhamnolipid (Rha1) with a high emulsification index (E_24_) of up to 80% against benzene, demonstrating its strong biosurfactant-producing capability. Similarly, *Pseudomonas* sp. strain AHE16, a plant growth-promoting rhizobacterium (PGPR), produced rhamnolipid (Rha2) with an emulsification index of 77%, further highlighting its effective emulsifying potential. The E_24_ values observed in this study are notably higher than those reported by^24^, where *P. aeruginosa* strain BM02, isolated from the Amazon region, exhibited E_24_ values ranging from 54 to 60%. This comparison suggests that the rhamnolipids produced by strains ZF2MGHSO and AHE16 have superior emulsifying activity, supporting their potential utility in environmental and industrial applications.

Rhamnolipids (Rhas), a class of glycolipid biosurfactants primarily produced by *Pseudomonas aeruginosa*, are among the most extensively studied biosurfactants due to two contrasting factors. First, they exhibit relatively high surface activities and are produced in relatively high yields after relatively short incubation periods by a well-understood, easy to cultivate microorganism. Second, they function as virulence factors in *P. aeruginosa*, contributing to its pathogenicity. Consequently, many aspects of Rha biosynthesis have been intensively investigated, both for industrial applications and for controlling its effects in infections^12^.

Structurally, Rhas consist of a glycon portion connected by an O-glycosidic bond. According to^25^, the glycon portion is made up of one or two rhamnose moieties connected to one another by an a-1,2-glycosidic bond. Although in certain rare homologs it can be acylated with a long chain alkenoic acid, the 2-hydroxyl group of the distal (with respect to the glycosidic bond) rhamnose group usually remains free^12^. However, the glycon portion is mostly made up of one or two rhamnose moieties, or in rare instances, three^23^. β-hydroxy fatty acid chains are most often saturated; mono- or polyunsaturated ones are less prevalent. Their chain lengths vary from C8 to C16. These fatty acid chains are linked to each other through an ester bond formed between the β-hydroxyl group of the distal chain and the carboxyl group of the proximal chain^12^. Our findings align with this structural characterization. Furthermore, a comparison between Rha2 and our previously produced rhamnolipid, Rha1 (manuscript under review), revealed that both share similar structures, with the primary difference being a shorter fatty acid chain length in Rha1.

In this study, higher accumulation rates of cadmium (Cd) and zinc (Zn) were observed in plants treated with the two rhamnolipids. Moreover, the expression of *HaZIP1* was significantly upregulated in both the roots and shoots of plants grown in rhamnolipid-supplemented soil, suggesting that this transporter plays a key role in Cd and Zn uptake and translocation in sunflower. ZIP proteins are well-documented for their involvement in Cd and Zn transport in plants^19,26^. The observed increase in Cd and Zn concentrations, along with the upregulation of *HaZIP1*, aligns with previous findings indicating that *ZIP* genes are predominantly expressed in roots and are crucial for Zn uptake from the soil into plants^22^. Furthermore, *ZIP* genes are also known to participate in Cd absorption and translocation^20^, supporting the hypothesis that *HaZIP1* plays a dual role in Zn and Cd transport in sunflower.

Currently, there is no direct molecular evidence that rhamnolipid treatment directly upregulates heavy metal (HM) homeostasis genes, including those in the ZIP (ZRT, IRT-like Protein) family. However, several indirect mechanisms suggest that rhamnolipids may influence the expression of these genes. First, rhamnolipids enhance heavy metal bioavailability by chelating metal ions and increasing their solubility in the rhizosphere, leading to greater metal uptake by plants^27–29^. This increased uptake may **t**rigger a homeostatic response, inducing the expression of metal transporters to regulate internal metal concentrations^30,31^. Second, excessive metal accumulation can cause oxidative stress, leading to the generation of reactive oxygen species (ROS), which act as signaling molecules activating various stress-response pathways, including the upregulation of metal transporter and detoxification genes^32^. Additionally, rhamnolipids have been shown to influence phytohormone levels, such as abscisic acid (ABA), which regulates metal uptake and homeostasis genes^33^. Given these mechanisms, our findings strongly suggest that *HaZIP1* plays a critical role in Cd and Zn uptake in sunflower plants, potentially as part of a rhamnolipid-induced regulatory response. However, further studies are needed to confirm whether rhamnolipids exert a direct regulatory effect on *HaZIP1* expression through transcriptomic and proteomic profiling, as well as hormonal and ROS analyses.

Several studies have demonstrated the role of microbial surfactants in improving the remediation of heavy metal-contaminated soils^15,16,34^. We propose that rhamnolipids enhance the phytoremediation efficiency of sunflower plants by increasing the bioavailability of Cd and Zn, which in turn triggers the expression of *HaZIP1*, facilitating metal uptake and translocation. Given that *HaZIP1* is primarily involved in Zn homeostasis but also mediates Cd uptake, its upregulation in response to rhamnolipid treatment highlights its dual role in heavy metal acquisition. The interplay between rhamnolipid-enhanced metal solubility and *HaZIP1*-mediated transport supports the hypothesis that rhamnolipids indirectly modulate metal transporter gene expression by altering metal availability and activating plant homeostatic responses.

While these results highlight the potential of rhamnolipid-assisted phytoremediation, transitioning from controlled laboratory conditions to real-world applications presents several challenges. Soil heterogeneity, microbial interactions, and environmental factors such as pH, organic matter content, and metal speciation could influence the stability and effectiveness of rhamnolipids in field conditions^35,36^. Additionally, large-scale applications require cost-effective production methods and an assessment of the long-term environmental impact of rhamnolipid use, including potential ecotoxicity and degradation rates^37,38^. To address these challenges, future studies should focus on field trials under diverse soil and climatic conditions to evaluate the feasibility of rhamnolipid-assisted phytoremediation in large-scale settings.

Although this study focuses on Cd and Zn, future research should explore whether rhamnolipids exhibit similar metal-chelating effects and influence transporter gene expression for other heavy metals (e.g., Pb, Cu, Cr) and organic pollutants (e.g., hydrocarbons, pesticides). Additionally, gene silencing approaches (RNAi, CRISPR) and overexpression studies should be conducted to determine if ZIP transporters and other metal homeostasis genes are directly regulated by rhamnolipids. Such studies could provide deeper insights into the molecular mechanisms underlying rhamnolipid-enhanced metal uptake and accumulation.

Remarkable uptake and accumulation of Cd and Zn were achieved in *Helianthus annuus* cultivated in soil supplemented with 2% (w/v) rhamnolipids further validate the role of biosurfactants in phytoremediation. ZIP family proteins are responsible for transporting various metals, including Zn and Cd, into the cytoplasm, either from the extracellular space or from internal storage compartments. The observed upregulation of *HaZIP1* is strongly correlated with increased heavy metal uptake and accumulation. These findings support the potential of biosurfactant-assisted phytoremediation using sunflower plants as an effective, eco-friendly, and sustainable approach for remediating heavy metal-contaminated soils.

## Methods

### Bacterial strains and biosurfactant production

The bacterial isolates used in this study, *Pseudomonas* sp*.* strain AHE16 (OL862991.1)^13^ and *Pseudomonas aeruginosa* strain ZF2MGHSO (ON564984) (manuscript under review), were previously identified. The production of biosurfactant was achieved on Minimal salt medium composed of (g/L): NaCl (1.1), KCl (1.1), KH_2_PO_4_ (3.4), FeSO_4_·7H_2_O (0.00028), K_2_HPO_4_ (4.4), yeast extract (0.5), and MgSO_4_·7H_2_O (0.5). Two millilitres of a trace element solution containing (g/L): CaCl_2_·4H_2_O (0.24), ZnSO_4_·7H_2_O (0.29), MnSO_4_·7H_2_O (0.17), and CuSO_4_·5H_2_O (0.25) were added. The medium was further supplemented with 2% used vegetable oil as the sole carbon and energy source. After 72 h of incubation, cells were harvested, and the cell-free supernatant (CFS) was collected and stored at 4°C for further analysis.

### Evaluation of the biosurfactant produced from *Pseudomonas* Sp.

#### Emulsification index of cell-free supernatant

The emulsifying properties of CFS from both *Pseudomonas* sp. were assessed using the emulsification index (E_24_). In this assay, 2 mL of CFS were mixed with 2 mL of individual hydrocarbons (xylene, diesel oil, benzene, and engine oil) separately in glass tubes and vortexed for 2 min and allowed to stand for 24 h at room temperature.^39^ The height of the emulsion layer was measured using a ruler, and the E24 index was calculated as the percentage ratio of the emulsion layer height to the total height of the mixture. The positive and negative controls were SDS (1% w/v) and distilled water, respectively.

#### Oil displacement assay

A thin layering coat of motor oil was spread on the surface of approximately 40 mL of distilled water, according to^40^. One millilitre aliquot of CFS was added to the center of the oil film in a 9 mm Petri dish, forming a distinct clear zone within two minutes. The diameter of the clear zones was measured using a ruler. The positive and negative controls were SDS (1% w/v) and distilled water, respectively.

#### Parafilm M test

Twenty-five μL of each CFS collected from both *Pseudomonas* sp. were placed on the hydrophobic surface of parafilm M^40^. Each droplet’s diameter was measured after one minute. The results were recorded as a scale from ‘−’ to ‘+++’ to indicate the degree of spreading, from partial to complete. SDS (1%w/v) served as the positive control, while distilled water served as the negative control.

### Extraction, purification and characterisation of biosurfactant

Our *Pseudomonas* sp. was cultivated under optimal growth conditions for surfactant production. Afterward, cell-free supernatants were acidified using 6 M HCl to pH 4.0 and left overnight at 4 °C to allow pellet formation. Finally, supernatants were mixed with an equal volume (1:1) of methanol-chloroform at room temperature to extract the crude biosurfactant.

Afterwards, one millilitre sample of the biosurfactant solution was injected into the Gas Chromatography-Mass Spectroscopy (GC–MS) apparatus. The analysis was performed using an Agilent Technologies 5890 gas chromatograph equipped with a split/splitless injector and a mass spectrometer detector (MSD), with helium as the carrier gas. The injection volume was one μL, the injector temperature was 250 °C, and the ion-source temperature was 280 °C. These details were reported by^41^. The GC–MS analysis was scheduled with a total running time of 90.67 min. By utilising both the NIST08 mass spectral database and references to their mass spectra, the peaks in the chromatograms produced by these studies were located.

### Rhamnolipid-assisted Phytoremediation

#### Seed germination and experimental design

Pure variety seeds of *Helianthus annuus* L. V120 were obtained from the Agricultural Research Center, Ministry of Agriculture, Giza, Egypt. The seeds were surface-sterilized in a 1–5% sodium hypochlorite (NaOCl) solution for 1 min and then germinated.

Soil samples contaminated with zinc and cadmium were collected and analyzed as detailed earlier in our previous work^13^. Quartz sand was first cleaned with 6 M HCl for 48 h, then neutralized to pH 5.5 by washing with water. It was subsequently dried at 25°C and baked at 180°C for 4 h. The collected soil was diluted with sand at a ratio of 1:3 (wt/wt) to improve aeration, enhance drainage, and ensure a more homogeneous distribution of heavy metals. The soil-sand mixture was autoclaved at 121 °C for 40 min to sterilize it, eliminating microbial influence on heavy metal uptake and accumulation. One kilogram of sterile soil-sand mixes was placed into plastic pots (20 cm in diameter and 15 cm in length). Seven-days old seedlings were transferred to experimental soil. Plants were irrigated with sterile distilled water and sterile solutions of the two tested surfactants. The three treatments were as follows: Sterile distilled water (0% surfactants) as control (C), 2% (w/v) solution of rhamnolipid produced from *Pseudomonas aeruginosa* strain ZF2MGHSO (accession number ON564984) (Rha1), and 2% (w/v) solution of rhamnolipid produced from *Pseudomonas sp.* strain AHE16 (accession number OL862991.1) (Rha2). Twenty-eight-day-old plants were harvested for various measurements. Fresh plant samples were stored at − 80 °C for RT-PCR analysis. Shoots and roots were oven-dried at 60°C until a constant weight was achieved, after which the plant dry weight (DW) was used to determine metal concentrations.

#### Elemental content of plant samples

Total metal concentrations (Zn and Cd) were determined using an Analytik Jena AG contrAA 300 High-Resolution Continuum Source Atomic Absorption Spectrometer (Flame and Hydride mode)^42^.

#### Real-time (RT)‒polymerase chain reaction (PCR)

Total RNA was extracted from *H. annuus* shoot and root samples under both control (C) and Rhamnolipids treatments (Rha1 and Rha2) using Direct-zol™ RNA MiniPrep Kits, followed by DNase treatment to remove genomic DNA. Complementary DNA (cDNA) was then synthesized from 1 ng of mRNA using the Invitrogen™ RETROscript™ Reverse Transcription Kit. RT-PCR primers were designed, and quantitative real-time PCR (qRT-PCR) was performed using the ABI PRISM 7900HT Sequence Detection System with SYBR Green (Thermo Fisher Scientific) as the fluorescent DNA-binding dye. Each reaction contained 10 µl of qPCR Master Mix, 5 pmol of primers, and 0.6 µl of SYBR Green at a final concentration of 1X. The thermal cycling profile was set to [2 min at 50 °C, 10 min at 95 °C, followed by 40 cycles of 15 s at 95 °C and 60 s at 60 °C]. A threshold reporter value (Rn) of 0.2 was used to determine the cycle threshold (CT)^43^. Relative transcript levels of *HaZIP* were calculated by normalization to *EF1α* as a constitutively expressed reference gene^32^. The primers were as follows: *HaZIP* (F-CCGGATGCTTTCGACCATCT and R-GCGATAAATCCTGCGAACGG). *EF1α* (F-TGCCCAAGAAGTTGCTGGTG and R-ACGTGCCCAGGTGAGTCGAT).

### Statistical analysis

All statistical analyses were conducted using R (version 2022.12.0 Build 353). Each assay included three biological replicates. Data are presented as mean values ± standard deviation (SD). Mean values were compared using two-way Analysis of Variance (ANOVA), with Tukey’s post-hoc test applied for multiple comparisons. A *p*-value of less than 0.05 was considered statistically significant.

## Data Availability

The datasets generated and/or analysed during the current study are available in the [GenBank] repository, [https://www.ncbi.nlm.nih.gov/nuccore/ON564984.1/], [https://www.ncbi.nlm.nih.gov/nuccore/OL862991.1].
